# A Novel Recyclable Magnetic Nano-Catalyst for Fenton-Photodegradation of Methyl Orange and Imidazole Derivatives Catalytic Synthesis

**DOI:** 10.3390/polym16010140

**Published:** 2024-01-01

**Authors:** Marzough A. Albalawi, Amira K. Hajri, Bassem Jamoussi, Omnia A. Albalawi

**Affiliations:** 1 Department of Chemistry, Alwajh College, University of Tabuk, Tabuk 71421, Saudi Arabia; ahejari@ut.edu.sa (A.K.H.); omniahayed@gmail.com (O.A.A.); 2 Department of Environment, Faculty of Environmental Sciences, King Abdulaziz University, Jeddah 21589, Saudi Arabia

**Keywords:** CDC@Fe_3_O_4_, Fenton-photocatalytic dye degradation, substituted imidazole derivatives

## Abstract

A magnetite chlorodeoxycellulose/ferroferric oxide (CDC@Fe_3_O_4_) heterogeneous photocatalyst was synthesised via treated and modified cotton in two steps. The designed nanocomposites were characterised by FTIR, TGA, XRD, SEM, and VSM analyses. The Fenton-photocatalytic decomposition efficiency of the synthesised magnetic catalyst was evaluated under visible sunlight using Methyl Orange (MO) as a model organic pollutant. The impacts of several degradation parameters, including the light source, catalyst load, irradiation temperature, oxidant dose, and pH of the dye aqueous solution and its corresponding concentration on the Fenton photodegradation performance, were methodically investigated. The (CDC@Fe_3_O_4_) heterogeneous catalyst showed a remarkable MO removal rate of 97.9% at 10 min under visible-light irradiation. (CDC@Fe_3_O_4_) nanomaterials were also used in a heterogeneous catalytic optimised protocol for a multicomponent reaction procedure to obtain nine tetra-substituted imidazole derivatives. The green protocol afforded imidazole derivatives in 30 min with good yields (91–97%) at room temperature and under ultrasound irradiation. Generally, a synthesised recyclable heterogeneous nano-catalyst is a good example and is suitable for wastewater treatment and organic synthesis.

## 1. Introduction

With the development of modern industry, the composition of industrial wastewater has become extremely complex, making water contamination highly problematic. Inappropriate wastewater treatment incorporating dyes can directly affect regional water quality, thereby threatening environmental and human health [1,2,3]. Of all the efficient treatment processes for organic colorants [4,5,6,7,8], the Fenton-type heterogeneous oxidation process involving Fe_3_O_4_ nanoparticles represents one of the most advanced and successful treatment technologies and has been extensively investigated for the removal of organic dyes with high efficiency coupled with the non-selective decomposition of organic pollutants [9,10,11,12]. Nevertheless, neat Fe_3_O_4_ nanoparticles exhibit a propensity to decompose in water. At the same time, Fe_3_O_4_ nanoparticles tend to aggregate owing to their anisotropic dipolar interactions, particularly in aqueous environments. This aggregation can result in reduced activity and dispersion of the nanoparticles [13,14].

A typical approach to deal with these disadvantages is to use support materials, including zeolite, porous silica, porous carbon, and carbon nanomaterials, to immobilise Fe_3_O_4_ nanoparticles [15]. However, most supporting materials have been found to be non-degradable or environmentally damaging. Moreover, their synthesis is complicated and involves the consumption of hazardous substances. Thus, it is crucial to develop a practical and straightforward approach for the production of biocompatible support catalysts with enhanced performance, stability, and recyclability over a broad pH range. This has been achieved owing to the availability of bio-based, recyclable, and environmentally compatible materials [16].

As an emerging class of biocompatible plant-derived nanomaterials, cellulose nanofibres (CNFs) have attracted considerable attention for wastewater treatment applications [17,18] because of their natural abundance, environmental compatibility, and high resistance strength [19] when used as a scaffold for magnetite nanoparticles. This technique can assist the homogeneous distribution of Fe_3_O_4_ to enhance the effective cross-linked specific surface area. Therefore, the catalytic efficiency increases. Associated research indicates that the magnetite agglomeration strategy can reduce magnetite agglomeration efficiently but only marginally enhances the catalytic activity of the obtained Fe_3_O_4_/CNF nano-catalyst in comparison to bare Fe_3_O_4_ nanoparticles [20]. Furthermore, the immobilisation of Fe_3_O_4_ nanoparticles onto substrates is essential to maintain their properties, preventing iron drainage during application in acidic environments. This leads to satisfactory durability and recovery properties of the catalyst. Biopolymers like chloro-deoxycellulose (CDC), alginate, cellulose, and chitosan can facilitate catalysis and offer numerous advantages, including low toxicity, cost-effectiveness, as well as high biocompatibility and biodegradability [21]. Recently, several researchers have addressed the advanced oxidation of bio-nanocomposite magnetic cellulosic fibres in aqueous solution using heterogeneous Fenton-like oxidation processes [22,23]. In another recent report, Wang et al. developed a new mussel-inspired magnetic carboxylated cellulose nanofiber (MCNF/PDA) for efficient Fenton-like methylene blue degradation [24].

Similarly, Multi-Component Reactions (MCRs) are accustomed to the standards of environmental chemistry aimed at reducing the generation of harmful waste [25]. Indeed, magnetically modified cellulose derivatives are gaining increasing interest in the field of organic synthesis because of the higher odds of increasing molecular structural diversifications [26,27,28]. Several recent studies have reported the use of new catalytic systems to produce a new range of molecules [29,30,31]. In recent years, imidazole scaffolds have been a vital class of important heterocycles due to their abundance in natural products and their extensive use in medicinal chemistry [32,33]. Several derivatives of imidazole are found within the structure of bioactive substances, encompassing anti-inflammatory [34], antidiabetic [35], antituberculosis [36], antifungal [37], antimalarial [38], and anticancer [39]. Various catalysts can be employed to synthesise imidazole. Common catalysts include transition metal catalysts such as palladium, platinum [40] or ruthenium complexes [41]. The use of palladium or ruthenium complexes for synthesising imidazoles is effective; however, it has some drawbacks. These transition metals, particularly palladium, can be costly, potentially increasing the overall synthesis expenses. Moreover, certain compounds of these metals may pose toxicity concerns, necessitating careful handling. The reaction conditions required for these complexes may require specific parameters, such as high temperatures or solvents, which could limit their versatility or complicate the process. In addition, the recovery and reuse of these catalysts can be challenging, potentially leading to increased waste generation or necessitating additional purification steps. Suboptimal selectivity might also be a concern, resulting in the formation of undesired by-products or requiring additional purification stages. Researchers often seek alternative catalysts or methodologies to mitigate these limitations and enhance the efficiency and sustainability of the synthetic process.

This study is the first to elucidate the design of a novel magnetic heterogeneous Fenton photocatalyst chloro-deoxycellulose/ferroferric oxide (CDC@Fe_3_O_4_) for degrading Methyl Orange (MO) organic dyes. The (CDC@Fe_3_O_4_) nanocomposites were synthesised using Fe_3_O_4_ nanoparticles embedded in chloro-deoxycellulose nanofibres. Therefore, we aimed to use (CDC@Fe_3_O_4_) nanoparticles to decompose MO under eco-friendly conditions. As a second application, (CDC@Fe_3_O_4_) was used as a recyclable nano-catalyst for an improved synthesis protocol of tetra-substituted imidazole. 

## 2. Materials and Methods

### 2.1. Materials

Cotton was furnished through SITEX (International Textile Society, Tunisia) and drabbed using hydrogen peroxide before magnetite nanoparticle coating. Methyl Orange, FeCl_2_·4H_2_O, FeCl_3_·6H_2_O, Thionylchloride (SOCl_2_) and NaOH, benzyl, 5-Chloro-salicylaldehyde, pyridin-2-amine, 5-methylpyridin-2-amine and 4-(tert-butyl) aniline are approved chemicals from Sigma-Aldrich (Steinheim, Germany).

### 2.2. Preparation of (CDC@Fe_3_O_4_)

**Scheme 1:**
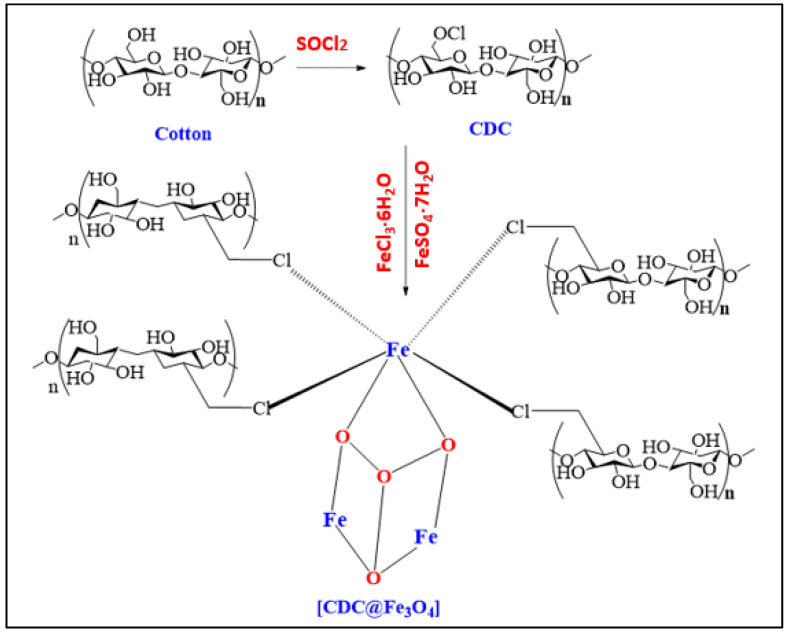
Synthesis of magnetic functionalised cotton.

Activated cotton (10 g) was activated at 80 °C for 12 h and dispersed in 200 mL DMF. Then, 35 mL of (SOCl_2_) was slowly added, and mechanical stirring was maintained at the same temperature for 4 h. Next, the cooled suspension of chloro-deoxy-cellulose (CDC) was washed several times with a dilute ammonium hydroxide solution. The pH of the supernatant was controlled to maintain the target natural pH, followed by suspension washing using distilled water. At the end of this step, the obtained samples were separated by filtration and dried at room temperature [42,43]. The coating of magnetic nanoparticles on chloro-deoxy-cellulose (CDC) was as follows: 100 g of Cotton, 3.0 g of FeSO_4_·4H_2_O and 7.5 g FeCl_3_·6H_2_O was dissolved in distilled water (200 mL of distilled water at 70 °C under mechanical stirring in an inert atmosphere. Next, a NaOH solution (4 M) was added dropwise to reach a pH of 12.0. The entire coprecipitation process was performed for 40 min. The precipitated magnetic nanocomposites were magnetically removed and cleaned using distilled water to obtain a neutral pH solution. The obtained nanocomposites (CDC@Fe_3_O_4_) were dried at 70 °C for 48 h (Scheme 1). 

### 2.3. Catalytic Synthesis of the Tetra-Substituted Imidazole Derivatives

To the solution of Benzyl Derivative (1.00 mmol), 5-Chloro-salicylaldehyde (1.00 mmol), and primary aromatic amines (pyridine-2-amine; 5-methylpyridin-2-amine; 4-(tert-butyl) aniline) (1.00 mmol) in protic solvent (Acetic acid, ethanol, water) (10 mL), 5 mg of the CDC@Fe_3_O_4_ nano-catalyst was added. The mixture underwent either reflux at various temperatures (80–120 °C) or ultrasound, and the reaction progress was monitored by thin-layer chromatography (TLC) using a mixture eluent (n-hexane: ethyl acetate 5:1). Upon completion of the reaction, the catalyst was separated using an external magnet and the reaction mixture was allowed to cool. Subsequently, the precipitate was collected by filtration, washed with water, and recrystallised using ethanol. All obtained products were characterised using FTIR, 1H NMR, and mass spectroscopy.

### 2.4. Apparatus

(FTIR) spectra were recorded on a Perkin Elmer spectrometer (Nicolet FTIR 460, 4000–400 cm^−1^ range, Waltham, MA, USA). (XRD) diffractograms were obtained using a Siemens diffractometer (D500, KR radiation, 15 KV, 1.5405 Å, FEI, Hillsboro, OR, USA). Thermogram curves (TGA) were obtained using Carl-Zeiss-Sigma (TA-SDT Q 600, New Castle, DE, USA). (SEM) micrographs were captured using a scanning electron microscope (SEM, Quanta-200, FEI Inc, Netherlands). The ^1^H NMR spectra of the synthesised compounds were recorded on a Bruker Avance 400 or 500 MHz NMR spectrometer (Billerica, MA, USA), using DMSO as the solvent. 

The magnetic properties of the catalyst were determined using a vibrating sample magnetometer (−1.7 to +1.7 range, Massachusetts, USA). Sonication was performed using an Elma-Ultrasonic device (S40, 800 WL^−1^, Elma Schmidbauer, GmbH, Singen, Germany). Absorption spectra were recorded using a spectrophotometer (SPECORD PLUS, 190–1100 nm, Analytik Jena GmbH, Germany). Colour changes in aqueous solutions were monitored in the 200−700 nm range to evaluate the removal efficiency during catalytic treatment. A 15 W Hg UV lamp was used for the preliminary Fenton photodegradation assays.

### 2.5. Photocatalytic Tests

Photo-Fenton experiments were conducted to assess the influence of the key operational parameters. These parameters included the initial quantity (5–35 mg) of the Fe_3_O_4_-based photocatalyst, MO concentration (5 × 10^−4^–35 × 10^−4^ M), initial pH_0_ (4–12), and H_2_O_2_ concentration (5–10 mmole·L^−1^). In batch trials, the procedure involved stirring with a magnetic stirrer and exposure to UV light or natural visible sunlight while controlling the reaction temperature by circulating water (25–45 °C). The experiments were conducted in an ultrasonic bath. Before initiating irradiation, the solution underwent a 90 min stirring period in darkness to ensure adsorption/desorption equilibrium of MO on the surface of the photocatalyst. During light exposure, samples of the reaction solution (3 mL) were extracted at regular intervals (0–15 min) and centrifuged to eliminate the catalyst. The degradation of MO was monitored by measuring the absorbance (A) at 464 nm using a UV–vis spectrophotometer. Subsequently, the percentage of degradation (%) was calculated.

## 3. Results and Discussion

### 3.1. Characterization

#### 3.1.1. UV-Vis Absorption Data

**Figure 1:**
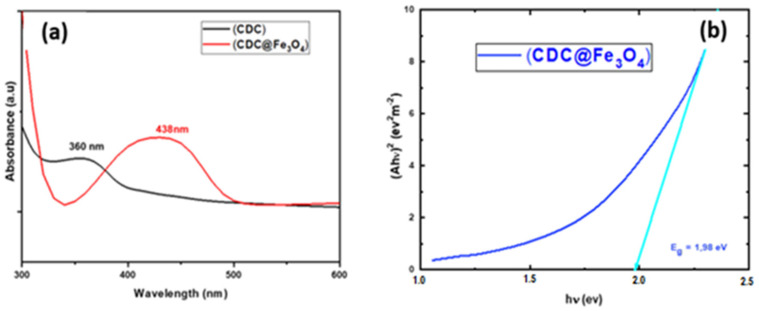
UV–Vis absorption of (CDC) and (CDC@Fe_3_O_4_) (**a**) and the corresponding bandgap data of (CDC@Fe_3_O_4_) (**b**).

Figure 1 depicts both the absorption spectra of (CDC) and (CDC@Fe_3_O_4_) as well as the recorded energy gap of the synthesized catalyst.

The designed photocatalyst and its corresponding ligand (CDC) showed maximal absorptions at ~360 and 438 nm, with absorption edges in the ranges of 320–380 and 340–500 nm, respectively, indicating that both synthesised materials are photolytically active in both the UV and visible regions. The bandgap energy of the magnetic catalyst is 1.98 eV (Figure 1b). A large range of light irradiation enhances the photocatalytic degradation performance. Numerous studies have reported that Fe_3_O_4_ nanoparticles exhibit direct band gap values ranging from 2.02 eV to 2.69 eV [44,45]. According to Deotale et al. [46,47], the bandgap of nanoparticles may be influenced by three factors: surface and interface effects, changes in the crystal structure due to heat treatment, and the Lattice strain within the sample.

The smallest band gap value observed for CDC–Fe_3_O_4_ (1.98 eV) can be attributed to the quantum size effect and surface effects arising from the differences in crystallite size. The larger crystallite size in CDC–Fe_3_O_4_:XRD (80 nm)/Fe_3_O_4_: XRD (5.1–10.03 nm) classifies them as p-type semiconductor materials when their optical gap energy is smaller than 3 eV.

Alternatively, it is plausible to propose that the lowest Eg value for CDC-Fe_3_O_4_ is not solely dependent on nanoparticle size, but also on their interaction with the chloro-deoxycellulose matrix in which they are embedded.

#### 3.1.2. FTIR

**Figure 2:**
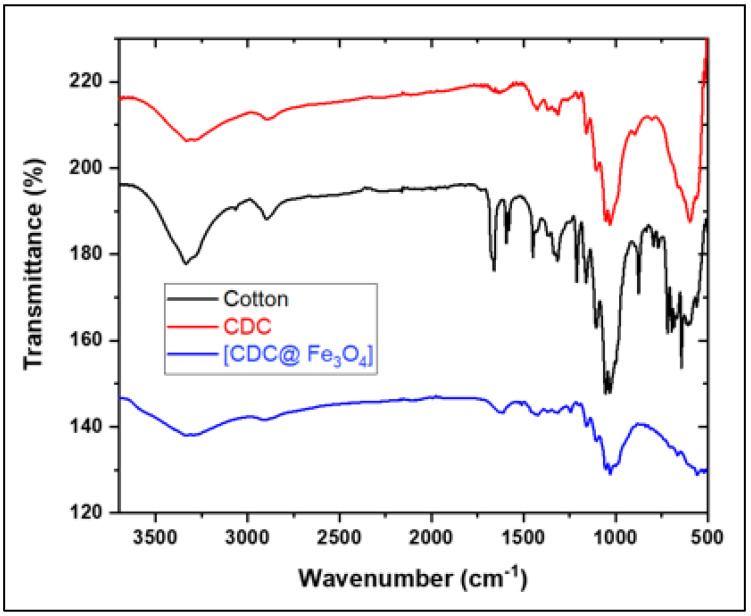
FTIR spectra of cotton, (CDC), and (CDC@Fe_3_O_4_).

The transmittance bands in the cotton graph (Figure 2) at 3300–3200 cm^−1^, 2906 cm^−1^, 1628 cm^−1^, and ~1020 cm^−1^ refer to the stretching vibrations of hydroxyl groups, stretching vibrations of CH, bending vibrations of hydroxyl groups, and C-O-C stretching vibrations, respectively [17].

FTIR spectroscopy was performed to analyse the corresponding target chemical structures induced by the modification of cotton fibres and coating with magnetite nanoparticles. Figure 2 depicts the graph of cotton (a), 6-CDC (b), and (CDC@Fe_3_O_4_) (c). The cotton spectrum shows the appearance of two characteristic bands around 883 and 1678 cm^−1^, ascribed to the stretching and bending vibrations of C–Cl, respectively [25]. These changes confirm the substitution of hydroxyl groups by chlorine atoms in (6-CDC) [25]. The intense band recorded at approximately 590 cm^−1^ is ascribed to the Fe-O stretching vibration [47]. This peak proves the successful coating of Fe_3_O_4_ nanoparticles on the modified cotton surface [48].

#### 3.1.3. TGA

**Figure 3:**
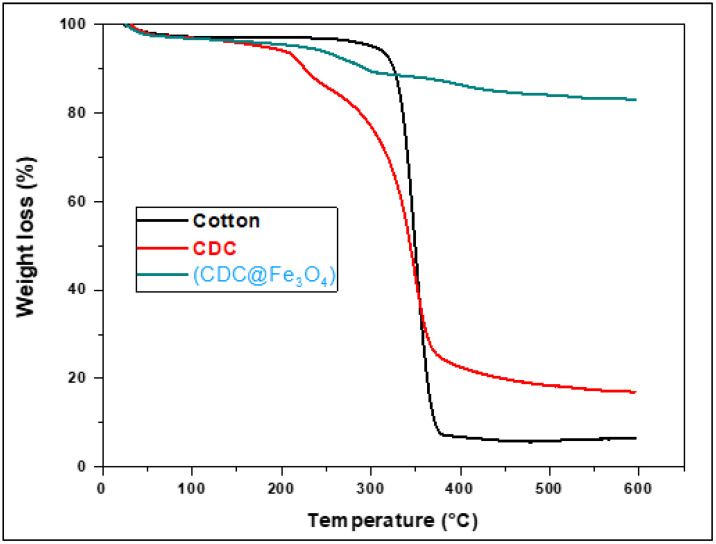
TGA curves of cotton, (CDC), and (CDC@Fe_3_O_4_).

The thermal stability of the synthesised materials was quantitatively evaluated using thermogravimetric analysis (TGA). Indeed, TGA is often used to determine the grafting density of the organic groups. The TGA thermogram of cotton (Figure 3) shows the first weight loss below 310 °C, corresponding to a relatively slow onset of degradation, whereas the major weight loss (93%) at approximately 320 °C may be ascribed to the degradation of glycosyl units.

Chlorine-modified cotton (6-CDC) showed relative mass stability up to 200 °C. Subsequently, the mass loss began at approximately 200 °C and extended to 382 °C, with one sharp weight drop and 16% remaining weight. The (CDC@Fe_3_O_4_) thermogram did not show significant mass loss up to 330 °C, and the remaining 83% of the total weight at 600 °C proposes that the synthesised magnetic nanomaterial satisfies the requirements of several applications that require elevated thermostability.

#### 3.1.4. VSM

**Figure 4:**
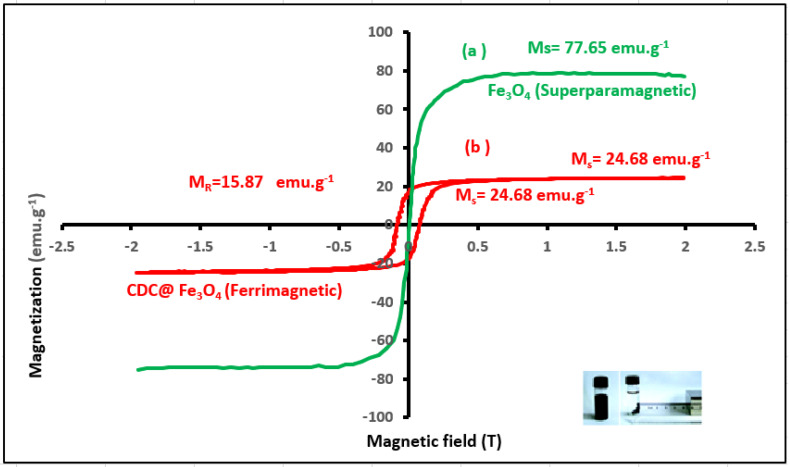
Magnetization versus applied field (M-H) curves for superparamagnetic Fe_3_O_4_ (a) and ferrimagnetic CDC@Fe_3_O_4_ (b).

The magnetization characteristics of (CDC@Fe_3_O_4_) magnetically modified cotton were investigated by recording the magnetic hysteresis loop (MH) at 300 K in addition to magnetization plots (M) versus an imposed magnetic field (T), as shown in Figure 4.

Based on Figure 4, the values of saturation magnetization (Ms) for Fe_3_O_4_ and CDC–Fe_3_O_4_ were measured at 77.65 and 24.68 emu g^−1^, respectively. Magnetisation examination provided evidence of the ferromagnetic properties of the designed nano-catalyst with detected remnant magnetization (MR) at 15.87 emu·g^−1^, and the matched coercive field (HC) reached 0.14 T. The difference between these saturation magnetization values can be attributed to the extinction of surface moments due to the functional groups of chloro-deoxycellulose serving as ligands around the Fe_3_O_4_ nuclei, occupying more volume and creating more physical distance between particles. The decrease in the magnetization can be understood as a consequence of some structural changes at the CDC@Fe_3_O_4_ interface, which resulted in the decrease of interactions between the spins at the surface of the nanoparticles. Accordingly, the obtained magnetic nanoparticles possessed excellent magnetic properties which suggest that they can prevent from aggregating and enable to redisperse rapidly when the magnetic field is removed. This result also implies that the functionalised magnetic nanoparticles have a lower magnetic quantity relative to magnetic nanoparticles Fe_3_O_4_.

According to Yazdani et al.’s [49] VSM hysteresis analysis of Fe_3_O_4_ nanoparticles synthesised via the co-precipitation method using various iron salts, the saturation magnetization (Ms) ranged from 30.5 to 53.38 emu/g. In the study by Deotale et al. [46], this value reached a maximum of 93.8 emu/g. Conversely, CDC@Fe_3_O_4_ exhibited a saturation value of 24.68 emu·g^−1^. The reduction in magnetic saturation can be attributed to the presence of the non-magnetic fraction, CDC, within the CDC@Fe_3_O_4_ composite. Figure 4 displays a notable feature of the emergence of coercivity field Hc, with a significant value of 0.13 T. This indicates a departure from the ferrimagnetic behaviour observed in the CDC@Fe_3_O_4_ particles compared to the superparamagnetic Fe_3_O_4_ nanoparticles. These observations can be explained by the surface effect and finite size effect. In general, smaller nanoparticles tend to exhibit a higher magnetic saturation than larger particles. This trend is due to the increased surface-to-volume ratio of smaller nanoparticles, which results in a greater number of surface atoms contributing to the magnetic moment. Thus, the VSM results confirmed the nanoparticle sizes found by XRD (88 nm) for the CDC@Fe_3_O_4_ system, aligned with the nanoparticle size results obtained by XRD, ranging between 5.1 and 10.03 nm as reported by Manikandan et al. [50].

#### 3.1.5. XRD

**Figure 5:**
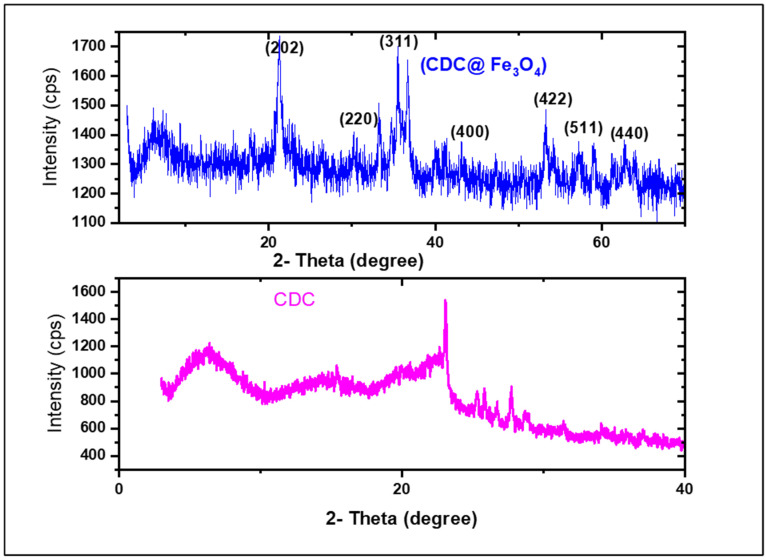
XRD patterns of (CDC) and (CDC@Fe_3_O_4_).

The crystalline structures of (CDC) and (CDC@Fe_3_O_4_) were registered using XRD examinations from 10 °to 50° in the 2θ scanning angle range, as depicted in Figure 5. The X-ray diffraction pattern of (CDC) displays an obvious main peak at approximately 2θ = 6.15°, 15.45°, and 23.06° corresponding to (1 1 0), (1 10), and (0 2 0).

The heterogeneous reaction of the ToS-Cl treatment can modify the polymer surface or its internal layers, thereby reaching the corresponding amorphous regions. The reduced intensity of the peaks indicates the presence of only small crystallites owing to the involvement of chlorine atoms in hydrogen bonds [51]. Such conduct reduces the space separating the chains, leading to peak shifting and providing higher h values. The confinement of the linked chlorine atoms enables them to form hydrogen bonds which can be distinguished from those formed at the surface of the material [52].

**Figure 6:**
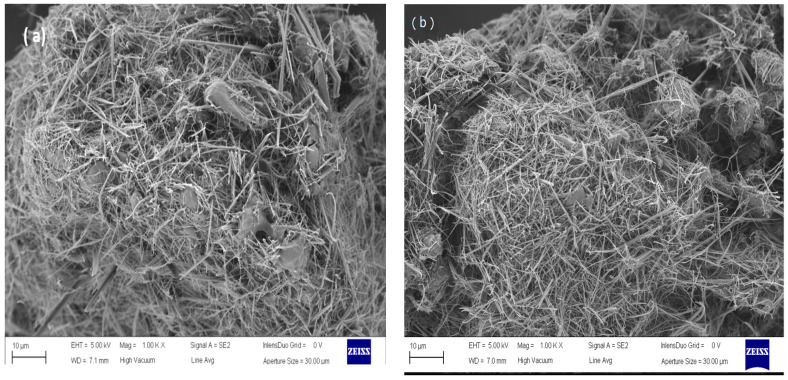
SEM images of (CDC) (**a**) and (CDC@Fe_3_O_4_) (**b**).

The XRD pattern of (CDC@Fe_3_O_4_) shown in Figure 6 shows a broad peak around 2ϴ = 15°, ascribed to the cellulosic moiety of the synthesised nanomaterial. The typical diffraction peaks of magnetite nanoparticles were observed at 21.22°, 30.42°, 35.52°, 43.14°, 53.35°, 57.34°, and 60.04°, corresponding to the (202), (220), (311), (400), (422), (511), and (440) crystal planes, correspondingly [53]. 

The average (CDC@Fe_3_O_4_) nanocomposite size was estimated to be 88 nm using the Scherrer equation:

D = K*λ*/*β* cos θ (1)

where D is the crystallite size, K is the crystallite shape factor (K = 0.94), k corresponds to the X-ray incident wavelength, *β* denotes the adapted FWHM referring to the full width at half maximum of the corresponding highest intensity recorded diffraction, and h symbolises the diffraction angle (2h = 35.52°) [25].

#### 3.1.6. SEM

Figure 6a,b shows SEM images of (CDC) and (CDC@Fe_3_O_4_), respectively. The surface morphology of both the synthesised compounds showed a fibrous network.

The arrangement of the iron oxide nanoparticles within the intricate three-dimensional network of (CDC) appears to be quite dense and organised. This unique fibrous structure appears to create an environment with specific pore sizes that could hinder the unhindered growth of magnetite nanoparticles during the coprecipitation process. As a result, what transpires is a phenomenon where the magnetite nanoparticles not only adhere to and cover the outer surfaces of the (CDC) material but also permeate through, occupying and filling the minuscule interfibrillar pores present within the fibrous matrix. This infiltration into the network suggests a comprehension integration of magnetite nanoparticles within the entire fibrous structure of (CDC), potentially influencing its properties and functionalities.

### 3.2. Optimisation of Photocatalytic Degradation Parameters

#### 3.2.1. The Effect of the Light Source on Fenton-Photodegradation Process

**Figure 7:**
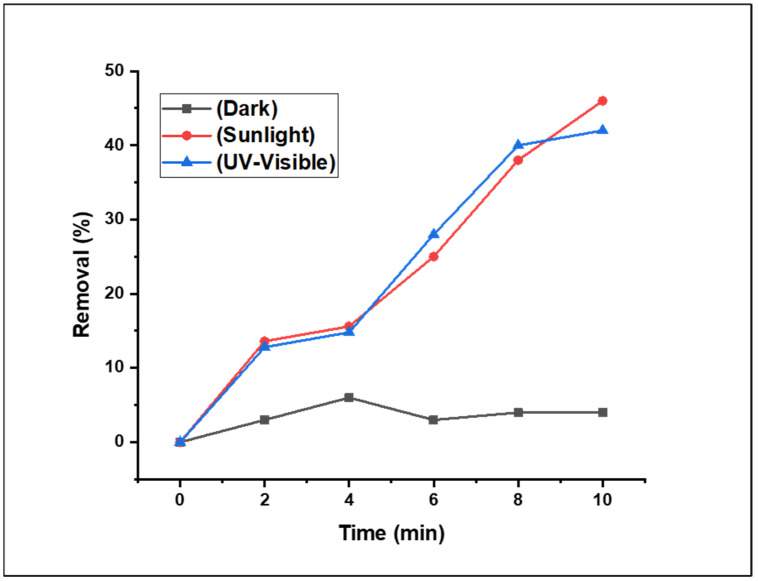
Variations in the MO removal rates under different light sources.

The synthesised (CDC@Fe_3_O_4_) nanocomposites exhibited Fenton-photocatalytic activity in both the UV and visible ranges, as verified in the UV–Vis spectrum. In this preliminary study, we introduced 5 mg (CDC@Fe_3_O_4_) and 5 mM of H_2_O_2_ to treat an aqueous solution (5 mg/L) of Methyl Orange (MO) at neutral pH and room temperature. Preliminary investigations were performed to highlight the effects of the light source on the removal efficiency of the designed photocatalyst. Thus, the Fenton-photocatalytic degradation study of MO dye using (CDC@Fe_3_O_4_) was carried out under three different conditions: in the dark and under visible and UV light irradiation. The results revealed that the (CDC@Fe_3_O_4_) nanoparticles were dynamic under both UV and sunlight (Figure 7).

As anticipated, the photodegradation results recorded under dark conditions may be neglected. In addition, the Fenton-photocatalytic tests performed under UV and natural light indicate that the removal of MO under natural solar light irradiation is noticeably more efficient than the treatment under UV light irradiation. Given the solar irradiation results, the synthesised (CDC@Fe_3_O_4_) photocatalyst successfully reduced 46% of MO, whereas the reduction attained under UV irradiation generated a 41% removal rate under the same degradation time (10 min), as depicted in Figure 7. From the above-mentioned data, it can be deduced that the degradation efficiency of MO under natural light is improved compared to the results obtained after UV irradiation. This improvement may be attributed to the concomitant existence of UV and visible light under natural solar irradiation [54]. Therefore, it can be concluded that the (CDC@Fe_3_O_4_) photocatalyst is active under sunlight, and the subsequent optimisation analyses are assisted by natural light.

#### 3.2.2. Preliminary Fenton-Photocatalytic Efficacity Studies of MO Reduction under Several Catalytic Systems

**Figure 8:**
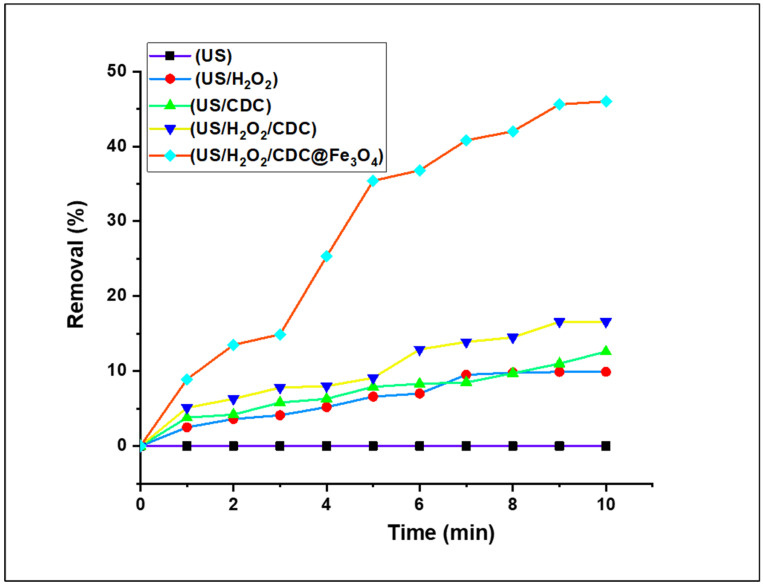
Variations in the MO removal rates under different catalytic systems.

As mentioned, the degradation of textile dyes is critical to devote effort for addressing environmental problems. Methyl Orange dye is among most used dyes in textile industries, which have ominous impacts on the environment. In this catalytic study, we investigated the effect of the treatment of aqueous solutions of these dyes using different combinations of catalytic systems: US, US/H_2_O_2_, US/(CDC), US/H_2_O_2_/(CDC), and US/H_2_O_2_/(CDC@Fe_3_O_4_) for this preliminary study, and 5 mg (CDC@Fe_3_O_4_) and 5 mM H_2_O_2_ to treat an aqueous solution (5 mg/L) of Methyl Orange (MO) at neutral pH and room temperature. All reduction assays were performed for 10 min. As illustrated in Figure 8, the aqueous solution of the studied dye solution was treated using a selected combination of catalytic systems. Apparently, the reduction of dye does not occur instantaneously and is to be monitored by referring to regular variations in the UV–Vis spectrum. An undetailed absorption study is presented in the following sections. As the exposure time of the desired reduced dye under the optimised catalytic system increases, the corresponding absorption peaks decrease. The reduction percentage and degeneration of Methyl Orange by the optimised catalytic systems progressively improved with time, thereby confirming the high reduction capability of US/H_2_O_2_/(CDC@Fe_3_O_4_).

A sequence of tests was conducted to evaluate the catalytic performance, and the results are shown in Figure 8. No removal rates were recorded using US alone as the catalytic system. It can be clearly observed that the decomposition rates using the US/H_2_O_2_ combination are very low (9.9% at 10 min), even with the assistance of H_2_O_2_. Using US/(6-CDC) and US/H_2_O_2_/(6-CDC) as Fenton-photocatalytic MO removal systems resulted in slightly improved degradation rates of 12.6 and 16.6%, respectively. The decomposition level increased to 46% upon the addition of [CDC@Fe_3_O_4_] to the solution. From these readings, it is evident that the (CDC@Fe_3_O_4_) nano-biomaterial has significantly enhanced Fenton-photocatalytic performance compared to that of (6-CDC). Accordingly, US/H_2_O_2_/(CDC@Fe_3_O_4_) was used in combination for further catalytic assays.

The efficiency of photocatalysis depends on the surface morphology, particle size, energy gap, crystallinity, and amount of hydroxyl radicals on the catalyst surface [55]. The generation of electrons and holes on the catalyst surface is followed by light absorption. They are then discharged or recombined to contribute to this reduction. In the case of providing an exterior surface for charge carrying, electrons and holes will displace. In this case, the generated electrons are entangled by the photocatalyst, whereas the holes are caught by hydroxyl radicals to generate HO_2_^•^ and OH^•^. The photocatalyst afforded an additional surface for the removal of charge carriers; hence, the formed hydroxyl radicals were used competently for the Fenton-photocatalytic decomposition of MO molecules. Moreover, hydroxyl radicals (OH^•^) are unstable and extremely dynamic chemical species that have an important impact on the Fenton-photocatalytic reduction. 

Degradation of MO by US/H_2_O_2_/CDC@Fe_3_O_4_ process is significantly higher than the degradation by US/H_2_O_2_/CDC and US/H_2_O_2_ processes. According to the results, the order of the degradation rate of MO has been determined as below: “US/H_2_O_2_/CDC@Fe_3_O_4_” > “US/H_2_O_2_/CDC” > “US/CDC”/” US/H_2_O_2_”> “US”. The chemical impact of the ultrasonic method is based on cavitation, in which cavities or bubbles are formed. These bubbles form at brief intervals, grow, expand, and collapse rapidly, thereby releasing substantial energy [56]. The collapse of these bubbles generates significant energy at the interface between the bubbles and liquid where the OH radicals are concentrated, thus promoting radical reactions within this zone [57]. •OH and •H radicals emerge during sonication of water [58,59], following steps (2)-(5):

H_2_O →))) •OH + •H (2)

•OH + •OH → H_2_O_2_ (3)

•OH + •H → H_2_O (4)

•H +•H → H_2_ (5)

The presence of •OH generated by the sonicator increased the number of radicals, thereby accelerating the rate of MO decomposition using the ultrasonic sound method compared to alternative approaches.

#### 3.2.3. Effect of (CDC@Fe_3_O_4_) Load on the Fenton Photodegradation Process

**Figure 9:**
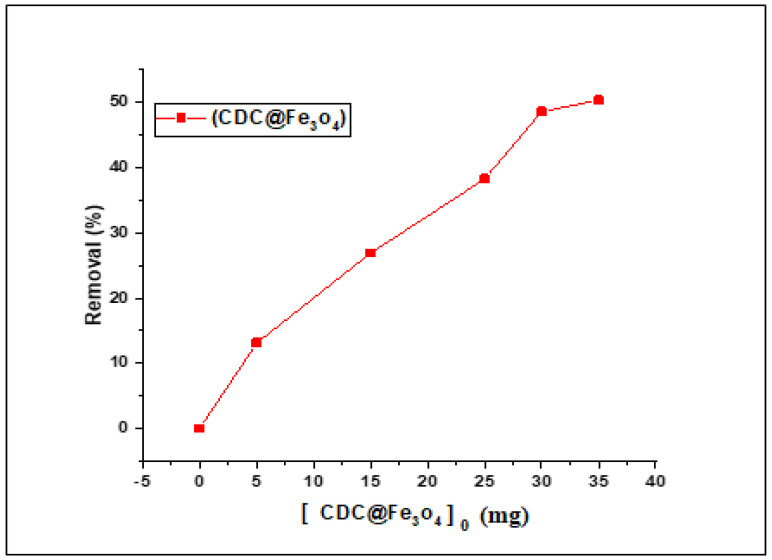
Effect of (CDC@Fe_3_O_4_) load on MO removal rate.

The catalyst load is a crucial factor in the dye removal process because the optimum dose of the catalyst provides more active sites and, thereby, efficient absorption of photons. To explore the impact of the magnetic photocatalyst load (CDC@Fe_3_O_4_) on the MO aqueous solution treatment, a series of five doses (5, 15, 25, 30, and 35 mg) was performed, and the findings are depicted in Figure 9. To determine the impact of catalyst load on dye reduction performance, an aqueous solution of methyl orange dye (5 × 10^−4^ mol L^−1^), was prepared and treated with the Fenton-photocatalytic system, US/H_2_O_2_/(CDC@Fe_3_O_4_), maintaining an initial dose of 5 mol L^−1^ of H_2_O_2_, neutral pH conditions, and a contact time of 10 min. It can be deduced from the figure that as the catalyst load increased, the MO degradation rate progressively increased. The degradation rate improved notably when (CDC@Fe_3_O_4_) amount was augmented from 5 mg to 30 mg. The highest MO removal rate (50.4%) was attained for (CDC@Fe_3_O_4_) (35 g), whereas a degradation rate of 48.6% was obtained using only 30 mg of the bio-polymeric photocatalyst.

Therefore, it was important to use a catalyst dose of 30 mg as the optimal amount for the photocatalytic degradation process. Generally, increasing the catalyst load leads to an increase in the number of surface-available active sites (Fe^2+^/^3+^ ions), and thereby, the dynamic generation of active radicals on the catalyst surface [60].

#### 3.2.4. Effect of MO Concentration on Fenton-Photodegradation Process

**Figure 10:**
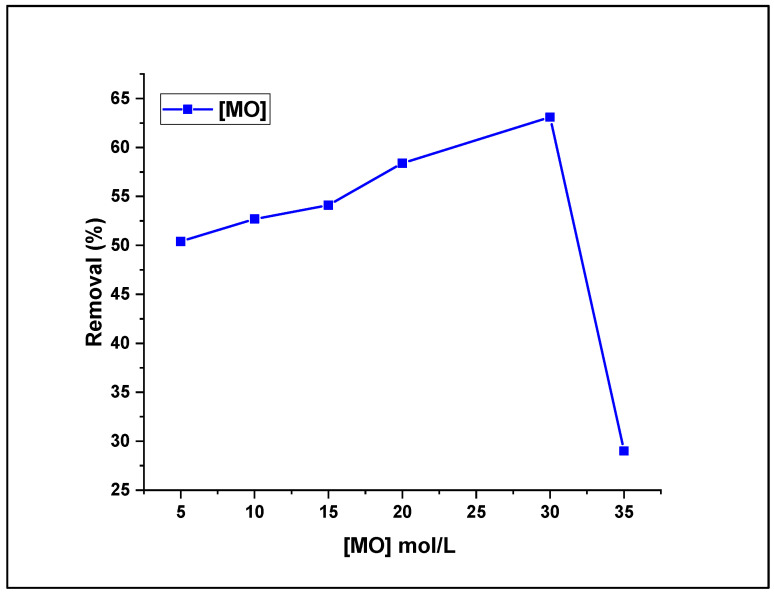
Effect of MO concentration on removal rates.

The impact of the initial concentration of the MO dye on the removal efficiency was also studied by increasing the dye aqueous solution concentration from 5 to 35 × 10^−4^ mol L^−1^ under sunlight and US irradiation, using a solution of 5 mM H_2_O_2_ and maintaining 30 mg as the catalyst dose at pH 7. The Fenton-photocatalytic removal results are graphically represented in Figure 10. 

The recorded data show that the degradation efficiency of (CDC@Fe_3_O_4_) seemed proportional to the treated dye concentration when the concentration of aqueous solutions was increased from 5 to 30 × 10^−4^ mol L^−1^, that is, the highest removal rate was 63.1% treating a 30 × 10^−4^ mol L^−1^ MO aqueous solution. In contrast, the lowest removal rate (29%) was recorded for the highest dye concentration (35 × 10^−4^ mol L^−1^). Upon increasing the MO concentration from 30 to 35 × 10^−4^ mol L^−1^, the photodegradation efficiency of MO decreased from 63.1% to 29%, as illustrated in Figure 10. This finding can be attributed to the fact that Fenton-photo-catalytically dynamic sites can be hidden with MO molecules which limit the light absorption and generation of radicals on the magnetic catalyst surface at increased dye amounts, thereby lowering the removal efficiency. However, photons easily reach the catalyst surface at inferior dye doses, and the formation of hydroxyl radicals is effortless [61]. Hence, the next Fenton-photocatalytic optimisation assays were carried out to reduce aqueous dye solutions with a concentration of which × 30 × 10^−4^ mol L^−1^.

#### 3.2.5. Effect of the Solution pH on the Fenton Photodegradation Process

**Figure 11:**
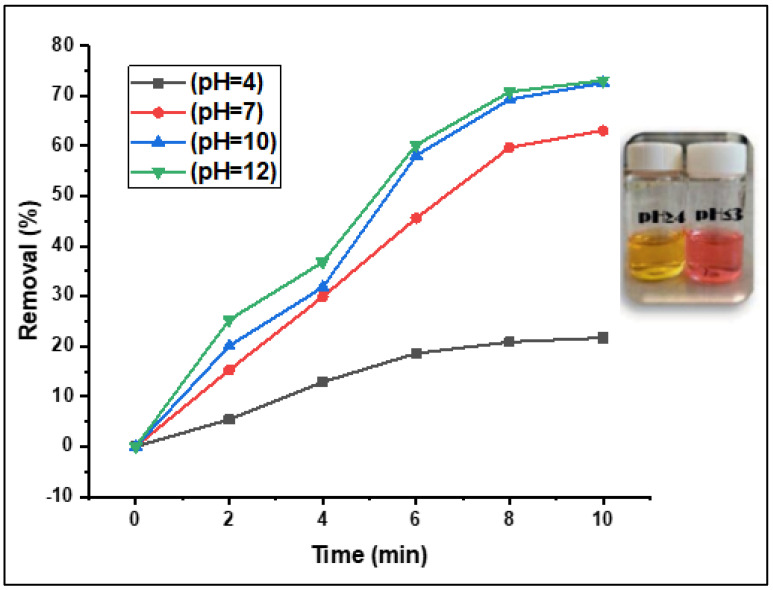
Variations of MO removal rates under different pH values.

As reported in several previous studies, the Fenton-photocatalytic performance of a catalyst is commonly associated with its capacity for the generation of hydroxyl radicals, thereby enhancing Fenton-photocatalytic removal by numerous folds at alkaline pH [62]. Figure 11 shows the effect of pH on the removal rate of the MO dye over the US/H_2_O_2_/(CDC@Fe_3_O_4_) Fenton-photocatalytic system. 

The effect of pH on the degradation of the MO dye was studied at several pH values (4, 7, 10, and 12) while keeping the aforementioned factors unchanged (i.e., 30 mg catalyst load, 30 × 10^−4^ mol L^−1^ concentration of aqueous dye solution, sunlight irradiation, and after 10 min of treatment). As predicted, the lowest removal rate (21.7%) was achieved at the minimum pH value (pH = 4). In contrast, the catalytic activity increased with increasing pH, and almost 73% degradation of MO was achieved at pH 10 and 12 (Figure 11). This result was possibly due to the increased rate of hydroxyl radical creation as well as their accumulation on the surface of the (CDC@Fe_3_O_4_) nanocomposites at high pH values [62]. Correspondingly, the chosen pH value for further Fenton-photocatalytic processes was 10.

#### 3.2.6. Effect of H_2_O_2_ Loading on the Fenton Photodegradation Process

**Figure 12:**
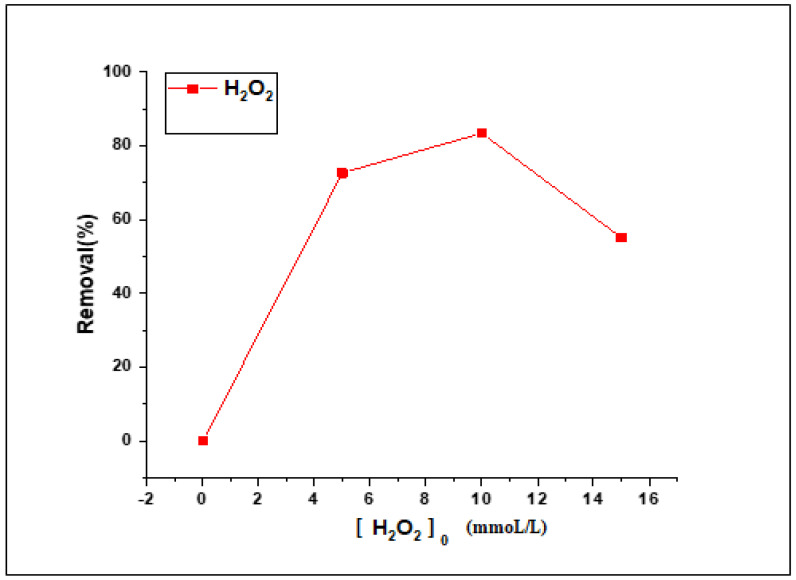
Effect of [H_2_O_2_] concentration on MO removal rate.

The concentration of H_2_O_2_ directly affected the removal efficiency of the Fenton photodegradation process. The impact of H_2_O_2_ dosage was investigated by estimating the oxidation process. The tested H_2_O_2_ loads were 5, 10, and 15 mmol L^−1^. Based on a similar study [63], we noticed that the use of high amounts of H_2_O_2_ leads to a “scavenger” impact of HO_2_^•^ radicals as well as HO^•^ radicals, which increases with increasing concentration. Therefore, the generation of radicals was partial [64,65]. In Fenton photocatalytic progression, the active radical scavenger species (HO^•^), superoxide radical anions (•O_2_^−^), and holes (h^+^) can be dissipated by hydrogen peroxide addition [65]. In this study, an amount of 10 mL of H_2_O_2_ was added (from the tested loads of 5 and 10 mmol L^−1^), which facilitates the refraining from the described scavenger impacts. The obtained findings displayed in Figure 12 show that MO Fenton photodegradation was enhanced swiftly by multiplying the concentration of H_2_O_2_ (5 to 10 mmol L^−1^) from 72.6 to 83.4%. The removal rate decreased to 55.1% with an increase in the concentration of H_2_O_2_ (10 mmol L^−1^). 

The decrease in the MO degradation percentage is attributed to the fact that an increased load of H_2_O_2_ results in a greater number of absorbed photons [66]. Moreover, more Fenton-photocatalytic sites are accessible, leading to a higher degradation rate. In contrast, attention must be paid because the use of the H_2_O_2_ fraction during treatment progression is suppressed, and therefore, an excessive quantity is not advisable. It has been stated in the literature that the presence of H_2_O_2_ is hazardous to a wide range of species and will considerably reduce the general decomposition rate if Fenton-photocatalytic oxidation is applied as pre-treatment for biological degradation. The detrimental effect of H_2_O_2_ is the scavenging of the generated hydroxyl radicals. This occurs when large volumes of hydrogen peroxide are applied [66].

#### 3.2.7. Effect of Temperature on Fenton Photodegradation

**Figure 13:**
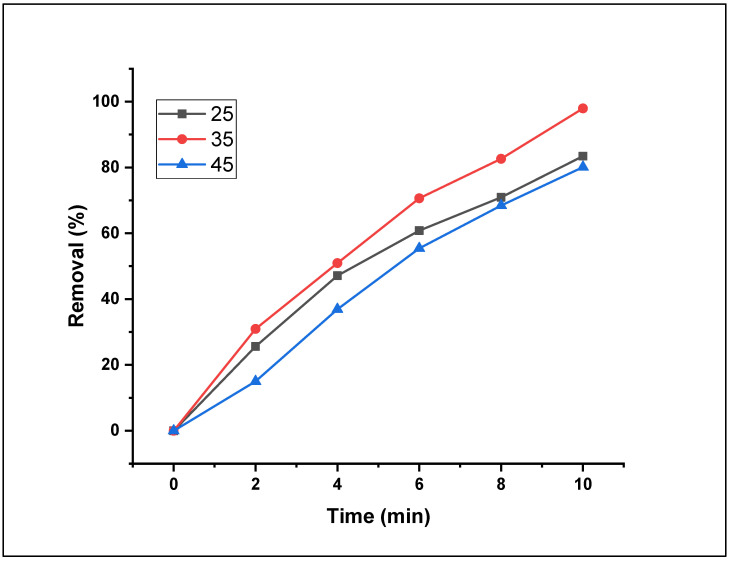
Effect of temperature on MO removal rate.

The evolution of the MO degradation level as a function of the reaction temperature was examined by incubating the aqueous dye solutions at various temperatures ranging from 25 °C to 35 °C to 45 °C. The elimination ratio of MO increased with increasing reaction temperature, from 83.4% at 25 °C to 97.9% at 35 °C. Figure 13 shows that the best temperature for the removal reaction was established when the reaction was performed at 35 °C. 

Indeed, increasing the temperature of the reaction mixtures led to a faster distribution rate of dye molecules in the nanomaterial. In this study, when the decomposition temperature was increased from 35 °C to 45 °C, the removal rate decreased to 80.1%. Comparable findings were reported by [67,68] when studying the impact of varying temperatures on dye-removal procedures. Nevertheless, adsorption trials are typically conducted at an adequate temperature to reduce operational costs. Thus, 35 °C was selected for the adsorption procedures in the present investigation.

### 3.3. Fenton-Photocatalytic Degradation of Methyl Orange: A UV-Vis Study and Proposed Fenton-Photocatalytic Mechanism

**Figure 14:**
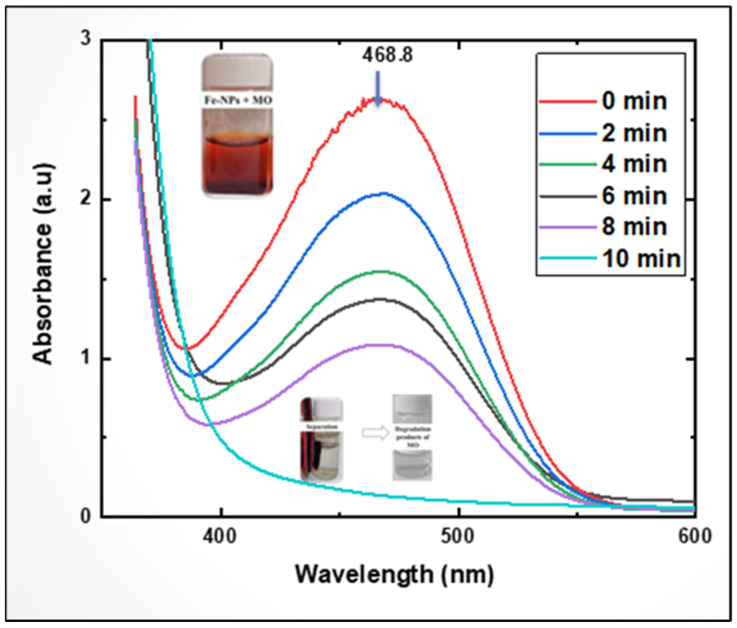
MO UV-visible spectra exhibited an absorbance decrease at 468.8 nm within 10 min. Photographs showing vanishing colour during the photocatalytic treatment.

The Fenton-photocatalytic performance of the (CDC@Fe_3_O_4_) photocatalyst was studied for the Fenton-photocatalytic decomposition of MO under sunlight. The UV spectrum of MO showed two clear absorption peaks at approximately 460 and 270 nm. The first peak can be attributed to the (–N=N–) chromophore azo bond, and the second peak at 270 nm to the MO corresponding to the benzene rings [69]. Figure 14 shows the gradual decrease in the intensity of the second absorption peak around 460 nm with increasing irradiation time. This finding shows that MO solution decolourisation by (CDC@Fe_3_O_4_) was achievable owing to the photocatalytic reactivity.

As methyl orange underwent photodegradation, the prominent 468.8 nm band linked to its azo bonds gradually diminished over time, giving way to an emergent peak at approximately 270 nm. This strongly implies generating degradation products or intermediates that exhibit light absorption at this wavelength. This alteration in the absorption characteristics signifies significant changes or degradation occurring within the molecular structure of methyl orange. The appearance of this 270 nm peak likely stemmed from the formation of smaller fragments or altered products resulting from the degradation process, possessing distinct light absorption properties compared to the methyl orange original molecule.

**Figure 15:**
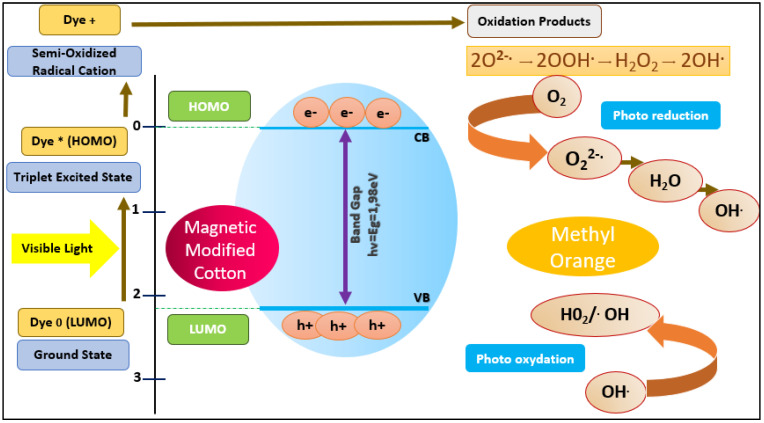
Proposed Fenton-photocatalytic degradation mechanism.

In general, the photocatalytic behaviour can be attributed to the association of both oxidation and reduction processes, where coated magnetite nanoparticles are active sites where the MO dye molecules are absorbed. The photocatalytic degradation mechanism (Figure 15) involves MO excitation under visible light from the ground state (MO 0) to the triplet excited state (MO*). Meanwhile, (MO*) produces semi-oxidised radial cations (MO•^+^) caused by electron injection into the magnetite conduction band. Hence, superoxide radicals (O_2_•^−^) are generated as a result of the response of the trapped electrons to the dissolved oxygen [70]. Consequently, these radical anions result in the generation of hydroxyl radicals (OH•) [71], which oxidise and degrade the target dye (Figure 15).

The immobilisation of iron nanoparticles on the modified surface of cotton fibres (CDC) did not affect the catalytic performance of iron nanoparticles. (CDC/Fe_3_O_4_) presents a network structure leading to an increase in the number of active sites available on the surface (Fe^2+^/^3+^ ions), leading to better charge carrier mobility owing to interconnected structures, a large specific surface area, and superior mass transfer performance. In addition, the modification of cotton fibres and coating with magnetite nanoparticles have the advantage of being reusable several times without difficulty, and upon comparing the degradation mechanisms of (CDC@Fe_3_O_4_) and Fe_3_O_4_, it becomes apparent that both processes involve the contribution of similar reactive oxygen species and radicals. Consequently, the degradation mechanisms exhibit a high degree of similarity. 

**Figure 16:**
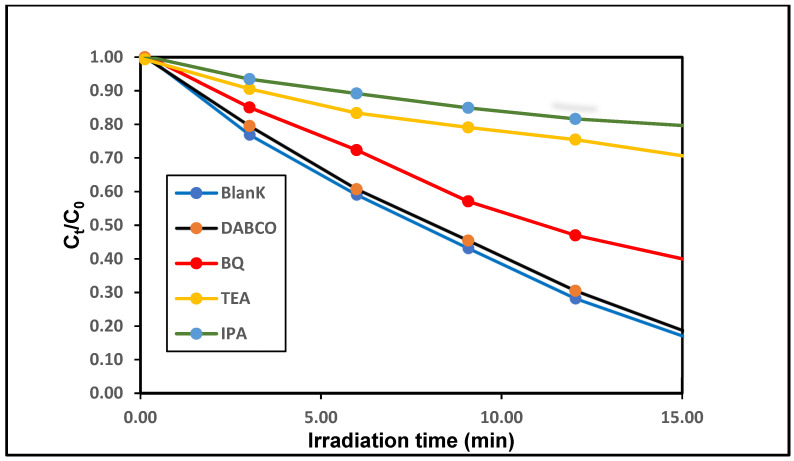
Varied effects of radical scavengers on MO degradation using US/H_2_O_2_/CDC @ Fe_3_O_4_.

Trapping experiments were performed to investigate the roles of the active species to further reveal the mechanism of the photocatalytic degradation of MO by the CDC@Fe_3_O_4_ nanocomposites under visible light irradiation. Different scavengers, such as triethanolamine (TEA, h^+^ scavenger), benzoquinone (BQ, •O_2_^−^ scavenger), isopropyl alcohol (IPA), •OH scavenger), and 1,4-Diazabicyclo [2.2.2]octane (DABCO; ^1^O_2_) [72,73], were chosen to evaluate their effects during the photocatalytic degradation processes when added separately. As shown in Figure 16, the primary active species involved in the photocatalytic degradation processes were •OH, h^+^, and •O_2_^−^.

Based on the above results and discussion, the chemical equations that reflect the mechanism of the photocatalytic degradation process are as follows:

CDC@Fe_3_O_4_ + hν → (CDC@Fe_3_O_4_) + h^+^ + e^−^ (6)

Fe^3+^ + e^−^ → Fe^2+^ (7)

Fe^2+^ + O_2_ → Fe^3+^ + •O_2_^−^ (8)

h^+^ + OH^−^ → •OH + H^+^ (9)

^1^MO + hν → ^3^MO* (10)

^3^MO + O_2_ → MO^•+^/M(-H^+^) + •O_2_^−^/HO_2_• (11)

•OH + O_2_^−^ + h^+^ + OM → degradation product (12)

### 3.4. Reusability of Fenton-Photocatalytic Performance of (CDC@Fe_3_O_4_)

The stability of heterogeneous photocatalysts is crucial, along with their performance, from practical application and economic viewpoints. In this investigation, heterogeneous photocatalyst nanoparticles were quickly removed from the catalytic system following degradation assays using an external magnet.

**Figure 17:**
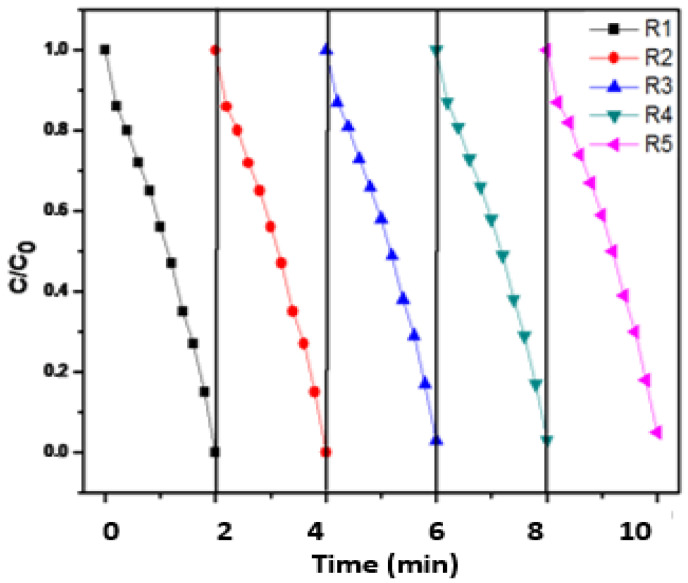
Five consecutive photocatalytic MO degradation processes of MO using (CDC@Fe_3_O_4_).

The removed catalyst was then collected and washed with distilled water. After exhaustive rinsing, the recovered photocatalyst was reused in the following run of MO removal under the optimised conditions five consecutive times. The results are shown in Figure 17. The photocatalytic performance of (CDC@Fe_3_O_4_) nanocomposites decreased slightly in the first catalytic cycle (from 97.9% to 93.8%) and then to 82% in the fifth cycle (Table 1). We noticed a drop-in catalytic activity after the sixth catalytic reuse (61.6%). 

The time course of MO removal throughout the five photocatalytic cycles under the optimised conditions is shown in Figure 17. The values of the constant rate for the corresponding first efficient cycles were 0.0978 min^−1^, 0.0987 min^−1^, 0.0984 min^−1^, 0.0978 min^−1^, and 0.0972 min^−1^. No major loss of catalytic activity for MO degradation was observed over (CDC@Fe_3_O_4_), proving that the synthesised magnetic nanocomposites possess good long-term stability and reusability. Thus, the synthesised magnetic photocatalyst appears to be promising for practical economic applications.

### 3.5. Theoretical Adsorption Kinetics Models

Theoretical adsorption models were evaluated to characterise the kinetics of (CDC@Fe_3_O_4_). Therefore, three theoretical models were selected based on the experimental results of the decolourisation process. Pseudo-first and -second order, as well as Elovich and intraparticle diffusion models, have been used to illustrate and adjust the parameters of kinetics adsorption. The equations governing these models have been described in various reports [74,75,76,77] 

**Figure 18:**
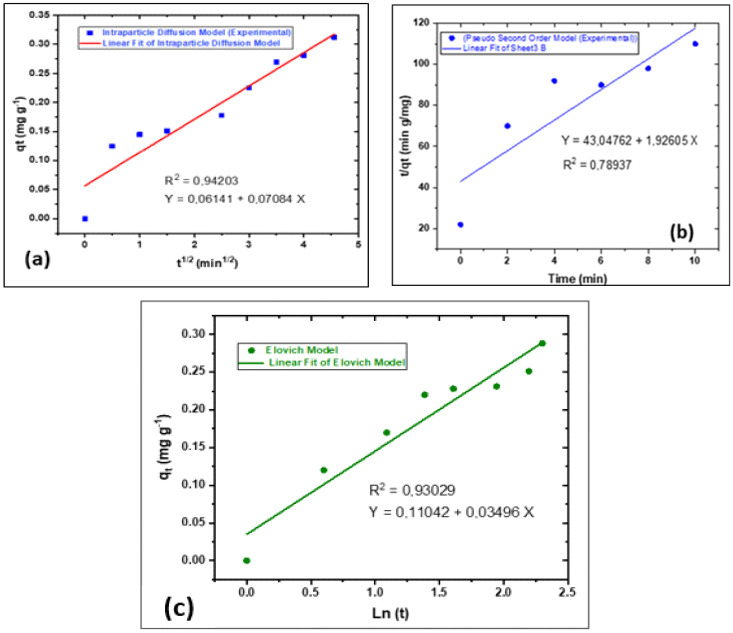
Investigated and simulated chosen kinetic adsorption models: (**a**) intraparticle diffusion, (**b**) pseudo-second order, and (**c**) Elovich.

Figure 18a–c show charts related to the kinetic adsorption models. As we can notice from these collected findings, the third model showed the maximal correlation rate, R^2^ (0.93029).

Based on this finding, it is possible to confirm that MO decolourisation treatment was performed along with intraparticle diffusion. The intraparticle diffusion model was mainly designated in three steps. First, immediate adsorption was detected because the external solution concentration was adequately elevated. Next, progressive adsorption behaviour was observed during the MO degradation process. Generally, the time required for this phase depends on the degradation parameters, including the catalyst load and its corresponding nanoparticle size, temperature, and aqueous solution concentration [78]. Finally, the degraded MO molecules exhibited moderate adsorption performance until the target equilibrium was reached. Thus, the intraparticle diffusion model explains the MO decomposition phenomenon.

### 3.6. Catalytic Synthesis of the Tetra-Substituted Imidazole Derivatives

**Scheme 2:**
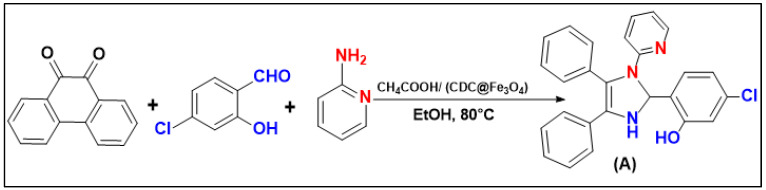
Schematic representation of imidazole derivative (A) synthesis.

To evaluate the efficacity of (CDC@Fe_3_O_4_) for the catalytic synthesis of nine 1,2,4,5-tetrasubstituted imidazole derivatives, the reaction of benzyl (1.00 mmol), (5-Chloro- salicylaldehyde (1.00 mmol), ammonium acetate (3 mmol), and pyridine-2-amine (1.00 mmol) was accomplished as the model reaction (Scheme 2).

The one-pot four-component reaction was first carried out to choose a suitable catalyst amount to obtain the highest amounts of 5-chloro-2-(4,5-diphenyl-1-(pyridin-2-yl)-2,3-dihydro-1H-Imidazole-2-yl) phenol (A). Table 1 presents the results. First, the rate of the chosen imidazole derivative (A) in the absence of (CDC@Fe_3_O_4_) after 12 h in acetic acid at 80 °C was relatively low (21%). Furthermore, the impact of catalyst loading was studied in subsequent tests (entries 2–7). Indeed, a loading of 3 mg of (CDC@Fe_3_O_4_) improved the yield of (A) in acetic acid at 80 °C for 12 h to approximately 46%. Next, the synthesis yield was tripled (63%) using 5 mg of the magnetic nano-catalyst (CDC@Fe_3_O_4_). However, no further improvement in the reaction yields was observed when using higher amounts of (CDC@Fe_3_O_4_) load (entry 7). In the next step, we aimed to eliminate the use of an acidic solvent and use a green solvent (entries 8 and 9). In addition, a similar study emphasised that solvent polarity is efficient for imidazole derivative synthesis [21]. Ethanol and water were tested as they are the most environmentally friendly solvents. The results in Table 2 indicate that the highest synthesis rate of (A) was attained in ethanol. Indeed, the polarity of ethanol and the high solubility of the starting reagents in this solvent, along with the generated hydrogen bonds between ethanol and water molecules issued from the synthesis reaction, are the main reasons for obtaining better yields in ethanol.

Subsequently, several experiments were performed to select the optimal energy output and reaction time (entries). As shown in Table 2, increasing both the reaction temperature and time did not significantly increase the reaction yields. By contrast, the synthesis of (A) under ultrasound irradiation increased the synthesis yield. The obtained reaction yield of (A) was 95% in ethanol using 5 mg of (CDC@Fe_3_O_4_) and only after 30 min of continuous ultrasound irradiation. 

Thus, the synthesis of the target imidazole derivatives was carried out under optimised conditions. Table 3 summarises the molecular structures and yields obtained in this study. 

**Scheme 3:**
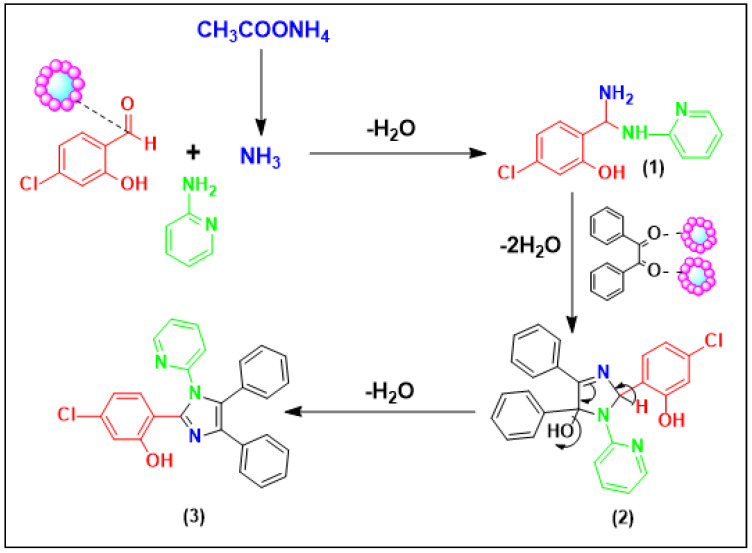
Proposed catalytic mechanism.

A probable catalytic mechanism is proposed for this Scheme 3. This mechanism is consistent with that of a previous report [79]. The scheme shows the proposed catalytic mechanism for imidazole derivative synthesis. First, intermediate (1) is generated following the nucleophilic addition of ammonia and the imine derivative to the initiated aldehyde. Afterward, we can postulate that the embedded Fe_2_O_3_ groups on modified cellulose are qualified as Lewis acid sites and initiate the carbonyl moieties by activating their corresponding oxygen atoms which produce a higher amount of intermediates (2), thereby increasing the generation of imine groups. Subsequently, the imine nitrogen atom breaks down the iminium moiety, thereby affording a related cyclisation along with dehydration, followed by reorganisation by hydrogen alteration to generate the target imidazole ring (3). It is significant to emphasise that all carried out catalytic reactions showed good yields despite their achievement under oxygen atmosphere. These findings indicate the non-oxidation features of the designed catalyst.

#### Reusability

(CDC@Fe_3_O_4_) can be easily removed using an external magnet. Subsequently, the recyclability probability was investigated. (CDC@Fe_3_O_4_) was cleaned using a cold water: ethanol mixture (2:1), filtered, and used in the next catalytic run. Table 2 shows the good yields of (A) throughout the five repeated runs. These results are ascribed to the excellent dispersion of the coating and non-agglomerated magnetite nanoparticles on the modified cellulose support. Table 4 shows that the developed values of both the turnover number (TON) and turnover frequency (TOF) of (CDC@Fe_3_O_4_) were 418.27 and 836.54 h^−1^, respectively.

**Figure 19:**
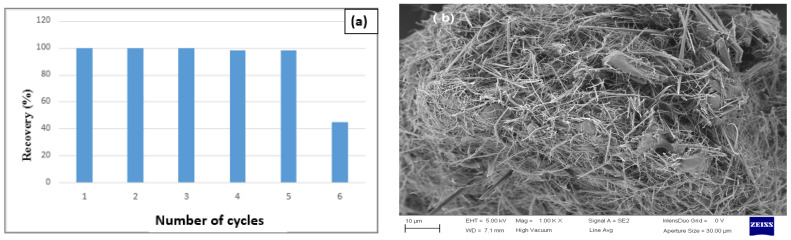
Efficiency of the catalytic run (**a**) and SEM images (CDC@Fe_3_O_4_) after six runs (**b**).

An exceptional decline in the synthesis rates was verified when carrying out the sixth catalytic run (Figure 18a). Figure 19b shows the discharge of most magnetite nanoparticles on the modified cellulose surface and, therefore, the decrease in catalyst efficiency starting from the sixth catalytic cycle.

## 4. Conclusions

In this report, we describe a simple two-step synthesis of a magnetically modified cellulose nanocomposite (CDC@Fe_3_O_4_). Characterisation of the obtained nanocomposites revealed the crystalline nature of the nanosized (CDC@Fe_3_O_4_) photocatalyst. The designed samples were used as heterogeneous catalysts for two applications. First, the designed catalyst was used for the photodegradation of Methyl Orange dye (MO). The (CDC@Fe_3_O_4_) nanocomposites were tested for Fenton-photodegradation of MO dye under dark, UV, and sunlight irradiation conditions, and it was assumed that the Fenton-photocatalytic process has a high photodegradation efficiency, which depends essentially on the variations in light sources, catalyst loads, dye concentration, treatment temperature, oxidant dose, and pH values of the treated dye aqueous solutions. The recorded kinetics of the Fenton photocatalytic process showed 97.9% degradation of MO dye in 10 min under the optimised degradation conditions. For the catalytic organic synthesis application, a low catalyst loading (5 mg) was used for an optimised multi-component reaction of nine tetra-substituted imidazoles in ethanol under ultrasound irradiation at room temperature. All derivatives were produced in good yields (90–97%). Moreover, all imidazole derivatives were easily isolated from the reaction mixtures. (CDC@Fe_3_O_4_) nanomaterial exhibited high catalytic activity after five consecutive runs without a remarkable decrease in yield. These findings provide new concepts for enhancing the Fenton-photodegradation catalytic efficacy of modified cellulose-based nanomaterials for improved applications in decontaminating organic dyes from water effluents and heterogeneous organic synthesis catalysis.

## Figures and Tables

**Table 1:** Removal rate during five consecutive catalytic cycles.

Cycle Number	Removal Rate
Cycle 1	97.9%
Cycle 2	93.8%
Cycle 3	90.3%
Cycle 4	85.4%
Cycle 5	82.0%

**Table 2:** Comparison of different catalytic systems.

Entry	Catalyst Load (mg)	Energy Output	Time	Solvent	Yield (%)
1	1	80 °C	12 h	Acetic Acid	21%
2	2	80 °C	12 h	Acetic Acid	25%
3	3	80 °C	12 h	Acetic Acid	46%
4	4	80 °C	12 h	Acetic Acid	50%
5	5	80 °C	12 h	Acetic Acid	63%
6	6	80 °C	12 h	Acetic Acid	63%
7	7	80 °C	12 h	Acetic Acid	63%
8	5	80 °C	12 h	Ethanol	75%
9	5	80 °C	12 h	Water	71%
10	5	120 °C	15 h	Acetic Acid	63%
11	5	US	30 min	Ethanol	95%
12	5	US/50 °C	30 min	Ethanol	95%

**Table 3:** Obtained tetra substituted imidazole derivatives under the optimised catalytic system.

Entry	Benzyl Derivative	Aldehyde	Primary Amine	Product	Yield (%)
A	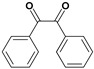	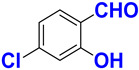	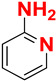	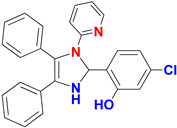	95%
B	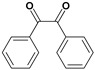	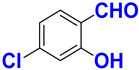	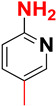	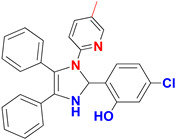	91%
C	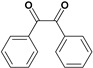	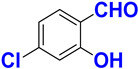	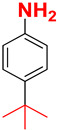	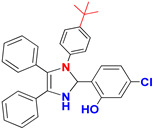	93%
D	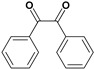	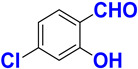	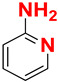	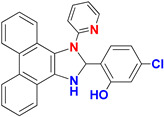	90%
E	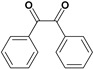	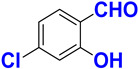	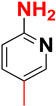	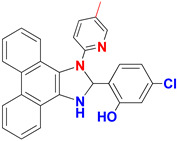	97%
F	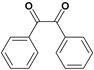	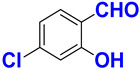	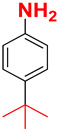	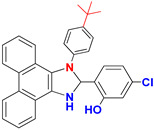	95%
G	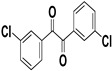	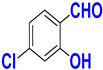	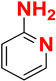	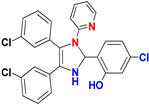	93%
H	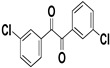	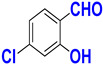	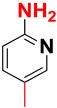	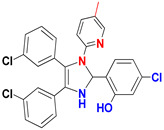	94%
I	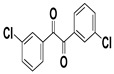	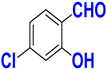	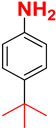	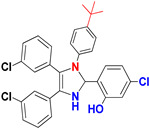	93%

**Table 4:** (CDC@Fe_3_O_4_) catalytic efficacity for six catalytic runs.

(CDC@Fe_3_O_4_) ^a^	TON ^b^	TOF (h^−1^) ^c^
Run 1	418.27	836.54
Run 2	398.75	797.50
Run 3	390.38	780.76
Run 4	384.81	769.62
Run 5	376.44	734.88
Run 6	223.08	446.16

^a^ Catalytic cycles were carried out six times to obtain (A) using the recovered amount of (CDC@Fe_3_O_4_) for 30 min. ^b^ TON = turnover number = total number of synthesised number of (A)/mole (CDC@Fe_3_O_4_). ^c^ TOF = turnover Frequency = n (A) synthetised per mole (CDC@Fe_3_O_4_)/hour at maximum synthesis yield = TON/time.

## Data Availability

The data presented in this study are available on request from the corresponding author.
